# Periodontal Disease and Environmental Cadmium Exposure

**DOI:** 10.1289/ehp.0901189

**Published:** 2009-12

**Authors:** Gianpaolo Guzzi, Paolo D. Pigatto, Anna Ronchi

**Affiliations:** Italian Association for Metals and Biocompatibility Research–A.I.R.M.E.B., Milan, Italy, E-mail: gianpaolo_guzzi@fastwebnet.it; Department of Technology for Health, Dermatological Clinic, IRCCS Galeazzi Hospital, University of Milan, Milan, Italy; Claudio Minoia, Laboratory of Environmental and Toxicology Testing, “S. Maugeri”-IRCCS, Pavia, Italy

We were pleased to see the article by Arora et al. (2009), which describes an association between environmental exposure to cadmium and periodontal disease.

In their cross-sectional study among U.S. adults, Arora et al. (2009) found periodontal disease in 15.4% of a nationally representative sample of 11,412 participants. The authors reported that for individuals with periodontal disease, as defined in their study, the geometric mean concentration of urinary Cd (0.50 μg/g creatinine) was significantly higher than for persons with no evidence of periodontitis (0.30 μg/g creatinine).

Arora et al. (2009) correctly stated that the main source of human exposure to environmental Cd is smoking. They proposed that additional sources of Cd in the general population are “emissions from industrial activities, including mining, smelting, and manufacturing of batteries, pigments, stabilizers, and alloys” (Arora et al. 2009).

However, in our view, one Cd source has been overlooked: intraoral dental alloys. Individuals with dental alloy restorations are regularly exposed to a number of trace elements that are continuously released from intraoral alloys (Wataha 2000).

Cadmium may be released from intra-oral alloys in dental patients and may be accumulated in both teeth and oral tissues, binding tightly to metallothioneins (Goyer and Clarkson 2001; Munksgaard 1992). For example, the intermetallic compound dental amalgam may contain approximately 4.5 μg/g Cd in the metal–matrix alloy (Minoia et al. 2007). Two metals other than Cd—lead (Dye et al. 2002) and mercury (Trivedi and Talim 1973)—probably contribute to periodontitis.

In a study of 268 avulsed teeth analyzed by atomic absorption spectrometry, Alomary et al. (2006) reported that the levels of Cd in tooth specimens were significantly higher in samples with dental amalgam fillings than in teeth with no amalgam. These findings suggest that exposure to Cd released from dental alloy restorations may influence many aspects of mineralized hard tissue of teeth and their immediate surrounding periodontal tissues. Another potential source of Cd is a metal dental bridge in which a Cd-containing alloy has been used for soldering.

In rare cases, Cd-containing dental alloys may lead to systemic intoxication (Borowiak et al. 1990). Even in dental acrylic-based resin for removable dentures, Cd might be used as a pigment.

It is therefore plausible that the release of Cd from both metal and/or nonmetal dental materials (i.e., resin-based materials) into the oral cavity may contribute to periodontal disease among adults.
